# Mental Health Mobile Phone App Usage, Concerns, and Benefits Among Psychiatric Outpatients: Comparative Survey Study

**DOI:** 10.2196/11715

**Published:** 2018-11-16

**Authors:** John Torous, Hannah Wisniewski, Gang Liu, Matcheri Keshavan

**Affiliations:** 1 Division of Digital Psychiatry Department of Psychiatry Beth Israel Deaconess Medical Center, Harvard Medical School Boston, MA United States; 2 Department of Biostatistics Harvard TH Chan School of Public Health Boston, MA United States

**Keywords:** smartphone, digital health, mobile phone, mental health, schizophrenia, depression, psychiatry, apps

## Abstract

**Background:**

Despite the popularity of mental health apps, it is unknown if they are actually used by those with mental illness. This study assessed whether differences in clinic setting may influence the use of mental health apps and which factors influence patient perception of apps.

**Objective:**

The objective of this study was to gain an understanding of how individuals with mental illness use their mobile phones by exploring their access to mobile phones and their use of mental health apps.

**Methods:**

A single time point survey study was conducted over a 2-week period in February 2018 at two nearby outpatient psychiatry clinics: one serving largely mood and anxiety disorder patients with private insurance staffed by both faculty and residents and the other serving largely psychotic disorder patients in a state Department of Mental Health (DMH) setting. A total of 25 patients at the state DMH clinic also consented for a single time point observation of apps currently installed on their personal mobile phone.

**Results:**

A total of 113 patients at the private insurance clinic and 73 at the state DMH clinic completed the survey. Those in the private insurance clinic were more likely to download a mental health app compared to the state DMH clinic, but actual rates of reported current app usage were comparable at each clinic, approximately 10%. Verifying current apps on patients’ mobile phones at the state DMH clinic confirmed that approximately 10% had mental health apps installed. Patients at both clinics were most concerned about privacy of mental health apps, although those at the state DMH clinic viewed cost savings as the greatest benefit while those at the private clinic reported time as the greatest benefit.

**Conclusions:**

High interest in mental health apps does not automatically translate into high use. Our results of low but similar rates of mental health app use at diverse clinics suggests DMH patients with largely psychotic disorders are as interested and engaged with apps as those in a private insurance clinic treating largely mood and anxiety disorders. Results from our study also highlight the importance of understanding how actual patients are using apps instead of relying on internet-based samples, which often yield higher results due to their likelihood of being selected.

## Introduction

### Unmet Needs

While there is clear potential for digital tools like mobile phone apps to increase access to care and services for mental health [1], less is known about the use of apps by patients. Interest in mental health apps is linked to increasing mobile phone access, with over 225 million people in the United States and over 2 billion people around the globe using these devices today [2]. There are already approximately 10,000 mental health and wellness apps available for immediate download [3], offering a myriad of services ranging from information to medication monitoring, coaching to telepsychiatry, and symptom tracking to support groups. But access and availability of mental health apps must not be conflated with safety, efficacy, or usability [4]. The majority of apps do not protect patient health data [5], have scarce evidence that they work [6,7], and are difficult to use and even harder to maintain longitudinal adherence with [8,9]. There are, of course, exceptions. Some research apps offer promise as useful clinical tools [10]. For example, one substance abuse app received FDA marketing approval in Fall 2017 [11]. In this evolving landscape of mental health apps, it is important to understand how end users, those diagnosed with and in treatment for mental illnesses, are actually using these apps and how they weigh the risks and benefits. This patient perspective is critical for informing patient-centered research and clinical efforts.

### Background

Like the rest of the world, those with mental illnesses have increasing access to mobile phones. The notion of a digital divide, that those with mental illnesses may not have interest in, ability to afford, or capability to use modern digital technologies, is no longer valid [12]. A 2013–2014 study of a first episode psychosis clinic reported 71% of patients owned a mobile phone [13], and a 2014 study of 320 psychiatric outpatients from four geographically distinct clinics around the United States reported 62% ownership [14]. Many Medicaid recipients in the United States may now qualify for a free mobile phone provided by the government [15]. With mobile phone ownership across the US population between 80% and 94% for those ages 18 to 29 years, these devices have become ubiquitous. Those with lower socioeconomic status and lower levels of income and education, likely to include many with psychotic illnesses treated at state Department of Mental Health (DMH) clinics, are more likely to be mobile phone–dependent, meaning they rely on their mobile phone as their primary means of internet access and communication [16].

However, access to a mobile phone does not mean a user will download health or mental health apps. The existing literature on app use in mental illness is rapidly expanding but still limited. There are high levels of interest among the general public who may screen at risk for mental illnesses based on online self-reported questionnaires, but those who are already online and volunteer to take internet surveys are likely a unique sample predisposed to favoring technology and apps. The nature of screening tests commonly used in these online surveys, such as the 9-item Patient Health Questionnaire (PHQ-9) for depression, makes generalization of results to those with diagnosed mental illness challenging. Research studies may also offer an inflated perspective on app use. A recent systematic review suggested that while use and adherence with mental health apps range between 44% and 99% in research settings, actual rates in real-world settings may range between 1% and 29% [17]. Still, case reports suggest that some patients are using mental health apps today [18]. In fact, during 2015 in the United Kingdom, 25% of National Health Service mental health trusts recommended mobile phone apps to patients [19].

Downloading a mental health app in itself does not mean it will be used or help achieve better mental health. Evidence suggests that most mental health apps are rarely used after being downloaded and only opened a few times [8]. For example, having access to a local gym, wanting to join that gym, having a gym membership, and actually going to that gym on a regular basis are all required to reap the benefits of the gym. Having access to a mobile phone, having interest in mental health apps, and downloading mental health apps are all necessary but not sufficient to guarantee regular mental health app use.

In this paper we seek to explore mental health patient access to mobile phones and their use of mental health apps. We aimed to capture the opinions and use from two groups of patients receiving psychiatric outpatient care: insured patients from a clinic primarily treating mood and anxiety disorders and state DMH patients from a clinic primarily treating psychotic disorders. To provide initial validation of self-reported app use, we also present results of mental health app use based on a count of the number and type of apps on the mobile phones of patients at the state DMH clinic.

We hypothesize that mobile phone ownership will have increased in both the private and state DMH clinic populations since our 2014 research but remain higher in the private clinic. We expect that a majority of mobile phone owners will have downloaded apps, but in both groups rates of mental health app downloads will be low and few people will report using mental health apps today. In verifying mental health app use today, we expect that self-report rates of app use will be similar to actual app use evidence on the phone itself.

## Methods

### Clinics

Two clinics sites conducted the survey. The first site was an outpatient psychiatry clinic serving insured patients for primarily mood and anxiety disorders. This clinic treats adults and sees approximately 1000 patients per month. The second study site was a state DMH outpatient psychiatric clinic that serves patients for primarily psychotic disorders. This clinic also treats adults and sees approximately 1000 patients per month. Both clinics are within one-half mile from each other in the urban environment of Boston, Massachusetts. Patients in either clinic are ineligible to be seen in the other.

### Surveys

**Figure 1:**
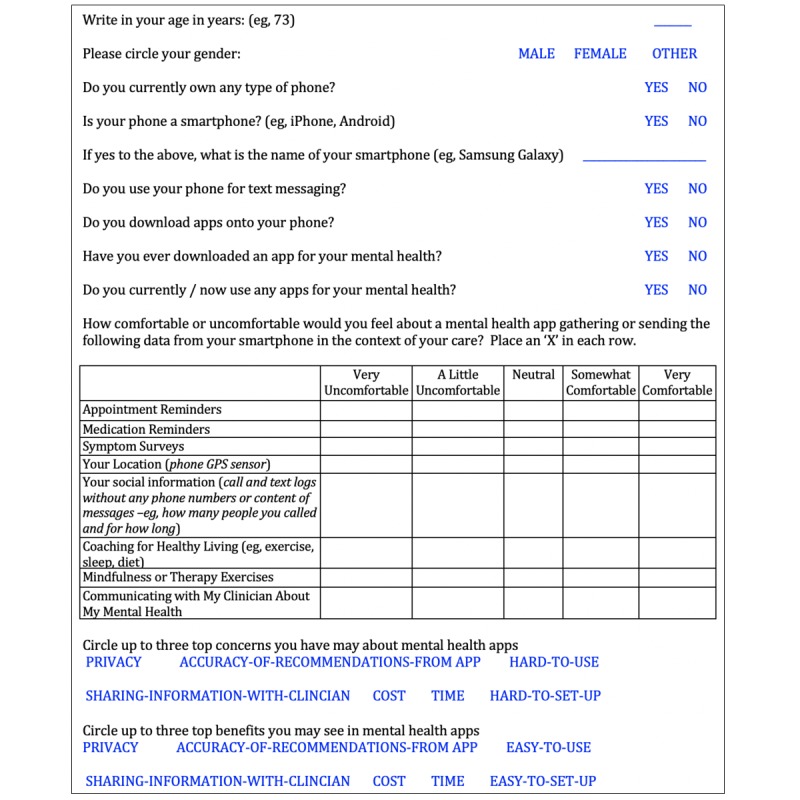
Survey.

Identical paper-and-pencil surveys assessing patient mobile phone ownership, use of apps, comfort with mental health app features, and perceived concerns and benefits were distributed to each study clinic. The survey was designed based on our prior similar research [14] as well as discussion and clinical experience with patients around their perceived benefits and concerns. Survey questions are displayed in  Figure 1. The surveys were made available to all patients in the clinic, who voluntarily completed them before or during appointments and submitted completed forms to the clinic staff. Surveys, along with handouts explaining the purpose, mental health focus, and voluntary nature of the study, were offered and provided to patients by clinic staff at both sites while patients were waiting for appointments. All surveys were completed in the clinic setting. All clinic patients were eligible. The survey was made available for 2 weeks at both study sites in February 2018.

Participants reported on comfort with features of mental health apps including appointment reminders, medication reminders, symptom surveys, passive data call and text log monitoring, passive data Global Positioning System (GPS) monitoring, coaching around healthy lifestyles (diet, exercise, sleep), mindfulness or therapy exercises, and communication with their mental health clinician. Results were recorded on a Likert scale: 1=very uncomfortable, 2=a little uncomfortable, 3=neutral, 4=somewhat comfortable, and 5=very comfortable. Results were stratified by mobile phone ownership, and significant differences in comfort between mobile phone and non–mobile phone ownership were calculated with a 2-sample *t* test.

### Phone Assessment

Patients at the state DMH clinic were eligible to opt in and have study staff record the names of apps on their mobile phone. For this part of the study, patients were asked to place their phone in airplane mode and allow study staff to write down the names of apps installed on their mobile phone. Because this is a novel methodology, we only examined the apps of 25 individuals as a pilot of the method.

Patients from the state DMH clinic were selected due to the lack of knowledge of app use in state DMH patients compared to private clinic patients [14]. In the future, we will expand this methodology to a greater number of individuals from both clinics.

### Analysis

Patients received no compensation or incentives to complete surveys but were paid US $20 to partake in the structured interview, which included recording the names of apps installed on their mobile phone. Patients had to own a smartphone to be eligible for this second part of the study. Recorded apps were organized by category according to their classification in the commercial marketplace at the time of the study. Results were entered into password-protected Excel spreadsheet software (Microsoft Corp), and all analyses and graphs were completed in the R programming language (R Foundation for Statistical Computing). Given the nature of the collected data, we applied descriptive statistics including *t* tests, chi-square tests, and other related methods. The institutional review boards at each of the study sites approved the study, and a waiver of informed consent was obtained for each site.

## Results

### Demographics

Of the estimated 500 patient visits to each clinic during the 2-week study duration, 113 patients completed the survey at the private clinic and 72 did so at the state DMH clinic, which is similar to prior completion rates in our 2013 survey. For the purpose of analysis, ages were bucketed into categories including 25 years and younger, 26 to 35 years, 36 to 45 years, 46 to 55 years, and 56 years and older. The mean age of patients in the state DMH clinic was 35.4 years and in the private clinic was 41.9 years. Other demographics and results of mobile phone and app ownership and use are presented in Table 1.

**Table 1:** Demographics and phone and app data.

Characteristics			Private clinic (n=113)		State DMH^a^ clinic (n=72)		*P* value
Gender, male, n (%)			62 (53.9)		32 (45.8)		<.30^b^
**Age (years), n (%)**							<.001^c^
	<25	5 (4.4)		13(18.0)			
	25-35	34 (30.0)		30 (55.6)			
	36-45	28 (24.8)		11(15.3)			
	46-55	27 (23.9)		13(18.0)			
	>56	19 (16.8)		5(6.9)			
Any phone ownership, n (%)			111 (98.2)		61 (84.7)		<.001^c^
Smartphone ownership, n (%)			102 (90.2)		48 (66.6)		<.001^d^
Downloaded apps, n (%)			99 (87.6)		36 (50.0)		<.001^d^
Downloaded mental health apps, n (%)			35 (30.9)		17 (23.6)		.28^d^
Used mental health apps, n (%)			11 (9.7)		7 (9.7)		>.99^d^

^a^DMH: Department of Mental Health.

^b^*t* test after *F* test to assess equal variance, *P*=.78.

^c^*Χ*^2^_14_=15.9.

^d^*t* test.

**Table 2:** Mobile phone ownership, app downloads, mental health app downloads, and mental health app use reported by state Department of Mental Health clinic patients.

Age group	Smartphone ownership, n (%)	Downloaded apps, n (%)	Downloaded mental health apps, n (%)	Currently using a mental health app, n (%)
<25 years (n=13)	12 (92)	11 (85)	4 (31)	2 (15)
26-35 years (n=30)	26 (87)	19 (63)	8 (27)	3 (10)
36-45 years (n=11)	6 (45)	3 (27)	3 (27)	1 (9)
46-55 years (n=13)	3 (23)	2 (15)	2 (15)	1 (9)
>56 years (n=5)	1 (20)	1 (20)	1 (20)	1 (20)

### State Department of Mental Health Clinic

**Figure 2:**
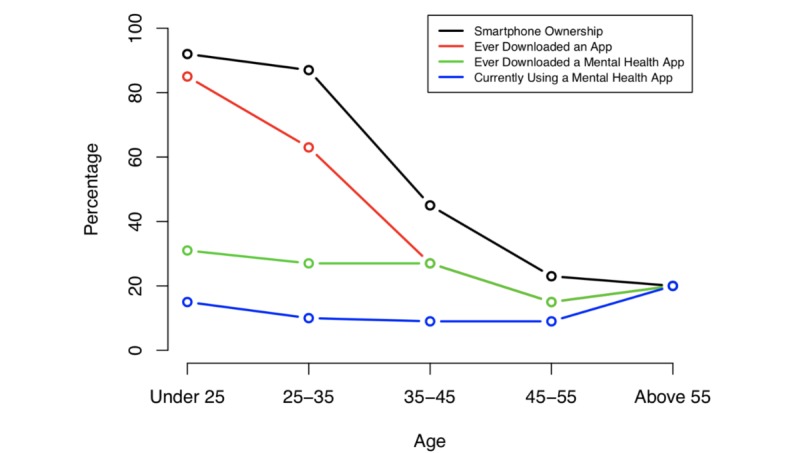
Mobile phone ownership, app downloads, mental health app downloads, and mental health app use reported by state Department of Mental Health clinic patients.

Percentage of mobile phone ownership in the state DMH clinic was highest among younger demographics, which mirrors national trends. However, mobile phone ownership did not guarantee interest in mental health apps. The overall prevalence of downloading a mental health app was 2.66 times lower compared to mobile phone ownership. Rates of downloading mental health apps were nearly equivalent over the first 3 age groups, suggesting interest is not limited to the youngest demographics. Roughly 1 in 6 patients at the state DMH clinic who reported owning a mobile phone also reported currently using a mental health app. Results are summarized in Table 2 and shown in Figure 2.

All features were found to be significant. Those who owned a smartphone reported more comfort with all features. The most discomfort was reported for passive data monitoring via GPS and call/text logs.

### Private Clinic

**Figure 3:**
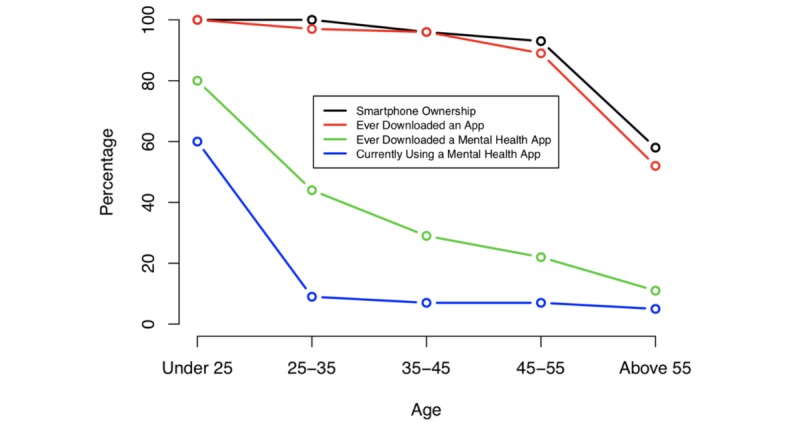
Mobile phone ownership, app downloads, mental health app downloads, and mental health app use reported by private clinic patients.

Percentage of mobile phone ownership in the private clinic was also highest among younger demographics, and mobile phone ownership did not guarantee interest in mental health apps. The overall prevalence of downloading a mental health app was 1.85 times lower compared to mobile phone ownership versus 2.66 times lower in the state DMH clinic. Downloading a mental health app did not guarantee active use today, which was 5 times lower compared to prevalence of download. Unlike in the state DMH clinic, there were higher rates of downloading an app among younger demographics. Mobile phone ownership was also higher overall compared to the state DMH clinic. Results are summarized in Table 3 and shown in Figure 3.

Those in the private clinic also reported higher levels of comfort with app features. Like at the state DMH clinic, a 2-sample *t* test found all features to be significant. As above, those who owned a smartphone reported more comfort with all features. The most discomfort was reported for passive data monitoring via GPS and call/text logs, which was also found in the state DMH clinic sample.

**Table 3:** Mobile phone ownership, app downloads, mental health app downloads, and mental health app use reported by private clinic patients.

Age group	Smartphone ownership, n (%)	Downloaded apps, n (%)	Downloaded mental health apps, n (%)	Currently using a mental health app, n (%)
<25 years (n=13)	5 (100)	5 (100)	4 (80)	3 (60)
26-35 years (n=30)	34 (100)	33 (97)	15 (44)	3 (9)
36-45 years (n=11)	27 (96)	27 (96)	8 (29)	2 (7)
46-55 years (n=13)	25 (27)	24 (89)	6 (22)	2 (7)
>56 years (n=5)	11 (19)	10 (52)	2 (11)	1 (5)

### Comparison Between Clinics

Apple and Android phones were not evenly distributed across clinic types. Apple phones were 3.5 times more prevalent in the private clinic compared with the state DMH clinic (49 vs 14), and Android phones were 1.55 times more prevalent in the state DMH clinic (34 vs 53). Lack of mobile phone ownership was 2.18 times more prevalent in the state DMH clinic (11 vs 34).

The number of app downloads also varied by clinic type. Those in the private clinic were nearly 3 times as likely to download an app compared with those in the state DMH clinic. But there was not a statistically significant difference by clinic type for currently using a mental health app, with both populations reporting approximately 9.7% use.

Reported comfort with mental health app features also varied by clinic. Those in the state DMH clinic reported feeling less comfortable with all features than the private clinic, except for passive monitoring. However, there was no statistically significant difference in comfort with passive monitoring with GPS and active monitoring with symptom surveys between clinics. All other differences were statistically significant from 2-sample *t* tests, as seen in Table 4.

**Table 4:** Comfort levels for mobile phone app features, measured with 5-point Likert scale and stratified by clinic type.

Feature	State DMH^a^ clinic	Private clinic	*P* value
Appointment reminders	3.82^b^	4.15^c^	.01
Medication reminders	3.31^b^	3.71^b^	.003
Symptom surveys	3.11^b^	3.50^b^	.06
Passive call/text monitoring	2.39^d^	2.32^d^	<.001
Passive GPS^e^ monitoring	2.78^d^	2.31^d^	.63
Coaching	3.1^b^	3.49^b^	.008
Mindfulness and therapy	3.17^b^	3.75^b^	.001
Communication with clinician	2.92^b^	3.54^b^	<.001

^a^DMH: Department of Mental Health.

^b^Neutral.

^c^Somewhat comfortable.

^d^Very uncomfortable.

^e^GPS: Global Positioning System.

**Figure 4:**
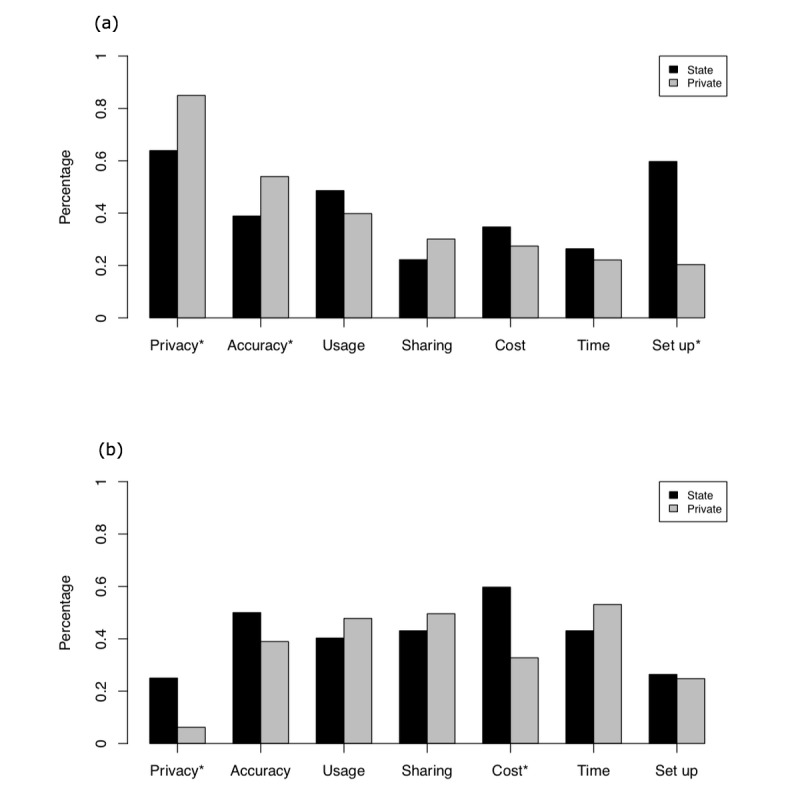
Concerns about and benefits of apps in the state and private clinics with (a) concerns and (b) benefits. Statistically significant differences are noted with an asterisk.

Patient concerns about mental health apps also varied by clinic, as shown in Figure 4 on the left. Across both clinics, privacy and accuracy were top concerns—although those in the private clinic reported significantly higher levels of concern for both. The setup and installation of mental health apps were viewed as the second highest concern among those in the state DMH clinic but the lowest concern among those in the private clinic. There was no significant difference in terms of usability, sharing data with clinicians, cost, or time. In terms of benefits, both groups reported similar responses. The only significant differences were between privacy and cost savings, as shown in Figure 4 on the right. Those at the state DMH clinic reported cost savings as the number one benefit of mental health apps and both groups reported privacy as having the lowest benefit from the choices presented.

### Validation of App Use

A total of 25 patients at the state DMH clinic allowed us to record the number and type of apps currently installed on their mobile phone. Of the patients who reported owning a mobile phone at the state DMH clinic, 17% (8/48) reported using mental health apps. Of the patients whose mobile phone apps were examined, only 12% (3/25) had actually downloaded mental health apps, providing preliminary validation for the self-reported data. However, in discussing mental health apps installed on the mobile phone, we learned that patients rarely used them. One patient explained he had installed a mental health coaching app but rarely used it, as he did not want to pay for the coaching features of the app. Another reported she had installed 2 mindfulness apps on her mobile phone at the urging of her social worker but rarely used them. A third had a symptom and medication monitoring app that he rarely used.

**Figure 5:**
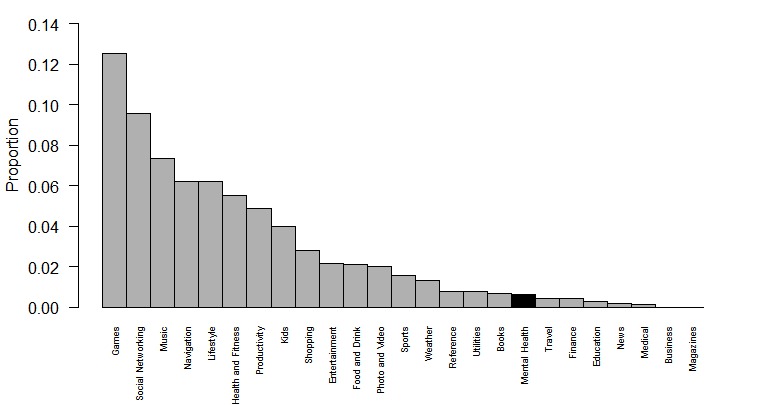
Mean count, by app category, of apps on the mobile phones of 25 patients in the state Department of Mental Health clinic. Although mental health apps are included in the health and fitness and medical categories, we added them as a separate category shaded in black for sake of comparison.

Thus, although these 3 patients did have mental health apps installed on their phones and used them occasionally, none appeared to be satisfied with or actively using them. Of interest, we found that 20% (5/25) of patients had installed horoscope apps (classified under lifestyle in Figure 5) and reported strong interest and engagement with these. While these patients may not have been actively using mental health apps, they did have many other types of apps on their phone, as reflected in Figure 5, which presents mean proportions of app categories. Health and fitness apps were the sixth most common mean proportion, and these largely represented step counter apps that patients reported using to track physical activity.

## Discussion

### Principal Findings

In this study, we surveyed 72 outpatients at a state DMH clinic treating largely psychotic disorders and 113 outpatients at a private clinic treating largely mood and anxiety disorders regarding access to and use of mental health apps. We found high rates of mobile phone ownership across both groups but lower rates of downloading and currently using mental health apps. In the state DMH clinic, the rate of downloading a mental health app was stable across the youngest 3 demographics, suggesting interest across a broader range of ages compared to the private clinic, where rates of downloads decreased in older age groups. After confirming mental health app use in the state DMH clinic, we found that self-reported app use was consistent but actual use, determined through discussion with patients, was less.

The potential of digital mental health tools like apps is fueled by their easy accessibility via mobile phones. In 2014, we reported that mobile phone ownership at the state DMH clinic was 49% [20] and at the private clinic 72% [4], and now their numbers have increased to 66% and 90%, respectively. But as seen in Figures 2 and 3, high rates of mobile phone ownership among those with psychiatric illnesses does not guarantee high rates of mental health app use. Even so, the 10% of patients who reported using a mental health app suggest bridging the new digital divide of app use has begun. The fact that reported app use did not differ between the private and state DMH clinics suggest patients’ early uptake of apps is not restricted to certain disease states or clinic populations. The fact that access to mobile phones was still lower at the state DMH clinic may reflect the lower socioeconomic status of this population, although our study was not designed to answer this question. But it does raise the issues that social determents of health likely impact digital health and there is a need to ensure equity in this evolving space.

A unique aspect of this study is the verification of reported mental health app usage by looking at the actual apps on the phones of a subset of patients. In the state DMH clinic, 9.7% of patients reported currently using mental health apps. Upon inspection of 25 patients’ phones, we found 12% had mental health apps installed, but when questioned the patients said they rarely used them. This raises the issue of how to accurately assess app use, as even objective measures of installed apps are not necessarily accurate. Although not the focus of our study, it is notable that health and fitness category apps were the sixth most prevalent type of app installed on patients’ mobile phones. The fact that games, social, media, music, and navigation represent the highest proportion of installed apps likely reflects that those with serious mental illnesses use their mobile phones in similar manners to the general public, as suggested in prior research [21]. It may be useful for future research to consider these top app categories and how features from these apps can be incorporated in mental health apps to improve uptake and use.

Our results suggest that patients have the most comfort with features such as appointment reminders and the least with passive data tracking features such as GPS and call/text log monitoring. Across both state and private clinics, those who owned a smartphone reported greater comfort for all features compared to those who did not. Those in the private clinic also reported greater comfort for all features compared to those in the state DMH clinic except for passive monitoring. However, the lack of any statistically significant difference for GPS tracking across both clinics and similarly low comfort scores for call/text monitoring represents a challenge to the often-posited advantage of mobile phone–based digital phenotyping [22]. While it is feasible today to gather sensor data from mobile phones and use this wealth of real-time data for a myriad of purposes, the privacy and ethical impacts of digital phenotyping are not lost on patients [23,24]. Outside of clinical studies, which involve volunteers who are compensated, will actual patients be willing to install digital phenotyping apps on their phones? On a more positive note, our results suggest that other features such as appointment reminders and app-based mindfulness or therapy exercises are likely to be better received. The popularity of psychic, not psychiatric, apps from our sample of 25 state DMH clinic patients also suggests that this class of app was popular in our sample.

Understanding the perceived benefits and concerns of mental health app users is necessary to ensure these tools are responsive to end user needs. Comparing both clinics, overall responses for benefits and concerns were similar. Although both groups felt privacy and accuracy were top concerns of mental health apps, those in the private clinic reported higher levels of concern for both. The state DMH clinic population reported higher levels of concern regarding difficulty setting up apps on their mobile phone. This suggests an opportunity to potentially increase uptake of apps in populations similar to our state DMH clinic sample by offering assistance in helping patients set up and install mobile phone apps. A technology navigator, a concept introduced by Ben-Zeev and colleagues [25], could fill this role and also offer information on privacy and accuracy of apps to help patients make more informed decisions around these chief points of concern. Comparing benefits across clinics, the fact that low cost was the top reported benefit among the state DMH clinic raises several issues for the digital health field. First, efforts to commercialize apps and charge fees could derail use among the most ill patients. This theory is buttressed by published case reports [18] and qualitative results from 1 of the 3 study participants who had a mental health app installed on her phone but reported lack of use because of cost. Second, although cost was seen as the top benefit and privacy a top concern, often the reason that an app may be free or low cost is because it is marketing or selling the users’ personal health information to third parties [26]. Thus, privacy and costs are tightly entwined in today’s health app ecosystem resulting in a paradox of both hindering and helping mental health app adoption.

Our results on mobile phone and app use are similar to recent reports. A 2016 survey of mobile phone ownership among those with mental illness offering peer support in New Hampshire identified that 58% owned a mobile phone, 61% had downloaded or used apps, and 72% use social media. This same study reported that 23% of these peers had used a mental health app [27], which is similar to our result of 23.6% in the state DMH clinic and 30.9% in the private clinic. The 2-year difference between studies may also explain higher rates of app adoption in our results. Our finding of high rates of mobile phone ownership in younger patients at the state DMH clinic (92% in those younger than 25 years and 87% in those aged 26 to 35 years) is similar to results from a study conducted in 2015 in a first-episode psychosis clinic where mobile phone ownership was 71% [13]. Again the 3-year difference between studies may help explain why our rates are higher. Our results that appointment reminder was the app feature with the highest reported comfort level is similar to a 2016 study of veterans receiving mental health treatment, also conducted in Boston, where appointment reminders were found to be the feature of highest interest [28]. Finally, another study surveying 82 mood and anxiety disorders patients in 2016–2017 regarding installing a mental health app found that just over 30% said they would be willing, which closely matches the 36% from the private clinic in our study who stated they have downloaded a mental health app [29].

### Limitations

Our study has several weaknesses that must be considered. While both clinics were within 1 mile of each other, both were also in a dense urban environment, suggesting our results may not be generalizable to rural settings. Like any survey study, there is concern for selection bias, although we note our rate of participation is similar to prior studies of this type [4,19,24]. Also, the self-reported nature of this study makes results difficult to verify, although our efforts to examine the actual apps installed on 25 mobile phones suggests our results are consistent with what apps are actually on patients’ phones.

### Conclusion

The potential of digital health to transform mental health requires more than access to mobile phones. Our results suggest that while mental health patients increasingly have access to mobile phones, far fewer are actually downloading and even fewer still using mental health apps. Bridging this new digital divide between access and use requires both understanding of the features patients want in apps as well as appreciating their concerns and desires. Tools like the American Psychiatric Association’s app evaluation framework can help guide informed decision making around selecting the right app for a patient’s needs—one that is safe, evidence based, engaging, and integrated into care [4]. To ensure these new digital tools remain useful to all patients and that the digital divide does not widen, we suggest continued efforts to look beyond internet-based samples of mental health app users and ensure that the perspectives of actual patients in care today are heard and acted upon.

## Abbreviations

DMH: Department of Mental Health
GPS: Global Positioning System
PHQ-9: Patient Health Questionnaire–9 item
